# Effects of *Lactobacillus brevis* additives on nutrient composition, fermentation quality, microflora structure and metabolites of *Pennisetum giganteum* silage

**DOI:** 10.3389/fvets.2025.1635386

**Published:** 2025-07-23

**Authors:** Juanrui Liao, Shaona Liu, Fuhua Yang, Yurong Fu, Hanqi Duan, Xiaowei Bao, Jinlong Huo, Zhiyong Zhao

**Affiliations:** ^1^Yunnan Academy of Animal Husbandry and Veterinary Sciences, Kunming, China; ^2^College of Animal Science and Technology, Yunnan Agricultural University, Kunming, China

**Keywords:** *Pennisetum giganteum*, *Lactobacillus brevis*, high-throughput sequencing, LC–MS, silage fermentation

## Abstract

The current situation of feed resource shortage can be effectively solved by high-value utilization of *Pennisetum giganteum*. This study investigated the effects of *Lactobacillus brevis* R-09 on *P. giganteum* silage quality, microbial structure and metabolites. In a randomized experimental design, silage was treated with (LT: 1.5 × 10^7^ CFU/kg *L. brevis* R-09) or without (LC: control) inoculant, each replicated six times. The LT group exhibited elevated crude fat (*p* < 0.05), lactic acid (*p* < 0.05), and isocaproic acid (*p* < 0.05) content, alongside reduced crude fiber, butyric acid, and mycotoxin levels (*p* < 0.05). High-throughput sequencing (16S/18S rDNA) revealed comparable microbial diversity across treatments, with Lactobacillus dominating the bacterial community. Notably, LT increased microbial uniformity and suppressed mold proliferation, diminishing their ecological impact. Liquid chromatography-mass spectrometry (LC–MS) profiling identified 464 differentially abundant metabolites, primarily linked to amino acid and lipid metabolism, suggesting a stabilized metabolic network. Taken together, these results suggest that the addition of *L. brevis* R-09 can improve the quality of *P. giganteum* silage by modulating microbial communities and metabolic pathways, thus providing mechanistic insights into the optimization of Lactobacillus-mediated silage fermentation.

## 1 Introduction

*Pennisetum giganteum* is a sustainable and high-quality feed source that provides a viable alternative to corn stover (1). Since its introduction to China in 1983 by Professor Zhanxi Lin of Fujian Agriculture and Forestry University, this African-origin perennial grass has undergone extensive domestication through more than two decades of systematic selection and agronomic research. The species has demonstrated remarkable adaptability, establishing successful cultivation across diverse climatic zones and soil types throughout China, with subsequent global adoption in over 30 Chinese provinces and 80 countries worldwide (2). Renowned for its exceptional agronomic characteristics - including rapid growth rates, high biomass yield, environmental stress tolerance, broad ecological adaptability, superior nutritional profile, and minimal invasive potential (3) - *P. giganteum* has become increasingly utilized in livestock nutrition (4). Alexandratos (5) predicts that global meat production will continue to rise between 2005 and 2050, with particularly notable increases in beef and lamb production, alongside a steady growth in milk production over the coming decade. However, the growing supply and demand for livestock products has led to a heightened need for feed resources, which has emerged as a significant constraint on the livestock sector (6). In this context, the use of *P. giganteum* as a silage resource offers significant advantages.

Silage, an ancient forage storage technology with a history of more than 3,000 years (7). Silage fermentation represents a complex ecosystem characterized by dynamic microbial interactions and metabolic processes (8). Within this ecosystem, *Lactobacillus* species emerge as key functional microorganisms, playing a crucial role in enhancing fermentation efficiency, improving aerobic stability, and suppressing pathogenic microorganisms through competitive exclusion and antimicrobial compound production (9). The application of high-throughput sequencing technologies has fundamentally transformed our understanding of silage microbial ecology, enabling comprehensive characterization of bacterial community dynamics throughout the fermentation process and precise elucidation of environmental factors shaping microbial succession patterns (10). Although previous investigations have employed these advanced techniques to analyze microbial community structures in various silage systems, including Sudan grass (11), wheat (12) and alfalfa (13), significant knowledge gaps remain regarding the microbial ecology and fermentation characteristics of *Pennisetum giganteum*-based silage, particularly in relation to lactic acid bacteria (LAB) supplementation. This study aims to establish an innovative pathway for the ecological and sustainable utilization of *P. giganteum* biomass resources through high-value silage production, addressing critical challenges in forage resource scarcity and feed grain reserve pressures in China.

## 2 Materials and methods

### 2.1 Silage preparation

*P. giganteum* was cultivated and harvested from the Niu Doduo Farming Professional Cooperative in Mengzi City (103°28′E, 23°18′N), located within the Honghe Hani and Yi Autonomous Prefecture of Yunnan Province, China. Mature plants, reaching approximately 2.5 meters in height, were harvested during the first cutting cycle and mechanically processed into 3 cm segments using a commercial forage chopper. The chopped material was supplemented with rice bran at 33% (w/w) of total fresh weight. Two experimental treatments were established: (1) control (LC), receiving no additive; and (2) 1.5 × 10^7^ CFU/kg *L.brevis* R-09 (provided by the Institute of Pig and Animal Nutrition, Yunnan Academy of Animal Husbandry and Veterinary Sciences, Kunming) treatment (LT). The control group received an equivalent volume of sterile distilled water. Following thorough homogenization, the material was compacted using a silage baler, with triplicate bags prepared for each treatment (n = 6 total). Ensiling was conducted at ambient temperature for 35 d, after which samples were collected for comprehensive analysis.

### 2.2 Chemical composition and fermentation quality analysis

Sample pH was measured using a calibrated portable pH meter (SI400, Spectrum Technologies, USA), while dry matter content was determined by oven-drying at 105°C to constant weight. Crude protein, fiber fractions (crude fiber, acid detergent fiber, neutral detergent fiber), and crude fat were analyzed according to Chinese National Standards GB/T 18868–2002, GB/T 20806–2022 and NY/T 1459–2022, with nitrogen content quantified using a Kjeldahl apparatus (K9860, Shandong Haineng Scientific Instrument, China). Aflatoxin (AFT) and T2 toxin (T2) mycotoxin levels were measured using commercial ELISA kits (Shanghai Enzyme linked Biotechnology Co, China), and short-chain fatty acids were analyzed by GC–MS (Agilent 8890B-7000D) following sample preparation involving freeze-drying, ultrasonication, and extraction with n-butanol containing 2-ethylbutyric acid as an internal standard. *P. giganteum* silage was evaluated and graded according to the “Standard for silage quality evaluation” issued by the Ministry of Agriculture of the People’s Republic of China, as shown in Supplementary Table S1.

### 2.3 High-throughput sequencing of *Pennisetum giganteum* silage

Total genomic DNA was extracted using the E. Z. N. A.® Soil DNA Kit (Shanghai Enzyme linked Biotechnology Co, China). Bacterial and fungal communities were amplified using specific primer sets: 16S rDNA (338F, 5’-ACTCCTACGGGAGGCAGCAG-3′ and 806R, 5’-GGACTACHVGGGTWTCTAAT-3′) and ITS (ITS1F: 5’-CTTGGTCATTTAGAGGAAGTAA-3′ and ITS2R: 5’-GCTGCGTT CTTCATCGATGC-3′), respectively. PCR products were purified using the AxyPrep DNA Gel Extraction Kit (Axygen, USA) and quantified with a Quantus™ Fluorometer (Promega, USA). Sequencing libraries were prepared using the NEXTFLEX Rapid DNA-Seq Kit (Bioo Scientific, USA) and analyzed on an Illumina MiSeq PE300 platform (Illumina, USA).

### 2.4 Bioinformatics analysis

Raw Illumina sequencing data underwent quality control and preprocessing using fastp (version 0.23.4) and FLASH (version 1.2.11) software, respectively. Operational taxonomic units (OTUs) were clustered at 97% similarity threshold using UPARSE version 7.1, with taxonomic classification performed against reference databases (RDP, Greengenes, MaarjAM) using the RDP classifier. Microbial community composition was analyzed at the genus level, while alpha diversity indices were calculated using mothur (version 1.30.1). Beta diversity analysis was conducted through Principal Coordinate Analysis (PCoA) based on Bray-Curtis distances, with statistical significance assessed using PERMANOVA. Microbial co-occurrence networks were constructed based on Spearman correlation coefficients (|r| > 0.6, *p* < 0.05), with network analysis and visualization performed using R (version 4.3.2) and Gephi (version 0.10.1).

### 2.5 Metabolite analysis

Metabolite extraction was performed on 50 mg silage samples using 400 μL ice-cold methanol: water (4: 1, v/v) containing 0.02 mg/mL L-2-chlorophenylalanine (internal standard). Samples were homogenized at −10°C (50 Hz, 6 min), ultrasonicated (5°C, 40 kHz, 30 min), incubated (−20°C, 30 min), and centrifuged (13,000 g, 4°C, 15 min). LC–MS analysis was conducted using UHPLC-Q Exactive HF-X (Thermo Fisher Scientific, USA), with data processed through Progenesis QI for feature extraction and metabolite identification against HMDB and Metlin databases. Multivariate analysis (PCA, OPLS-DA) was performed using the ropls R package, with significant metabolites selected based on VIP > 1.0 and *p* < 0.05 criteria. Pathway analysis was conducted through KEGG annotation and Fisher’s exact test.

### 2.6 Data processing and analysis

Data are expressed as mean ± standard error (SEM). Statistical analyses were performed using GraphPad Prism 9.0, with two-group comparisons assessed by Student’s t-test and multiple-group comparisons analyzed by one-way ANOVA. Statistically significant difference is indicated by *p* < 0.05, significant difference is indicated by *p* < 0.01, and highly significant difference is indicated by *p* < 0.001.

## 3 Results

### 3.1 Nutritional composition and sensory evaluation results

Following 35 days of ensiling, chemical analysis revealed significant alterations in silage composition: LT crude fiber content and pH values decreased significantly (*p* < 0.01), while crude fat content showed a marked increase (*p* < 0.01) (Table 1). The sensory evaluation results of silage are shown in Supplementary Table S2. The color of LT is closer to the original color of *P. giganteum*, without pungent sour smell, and the overall score and grade are better than LC.

**Table 1:** Chemical composition analysis.

Items	LC (g/kg DM)	LT (g/kg DM)	*p-*value
Dry matter	679.07 ± 26.12	687.85 ± 0.45	0.5913
Coarse ash	54.39 ± 5.14	56.47 ± 0.55	0.523
Crude fiber	295.97 ± 32.89*	238.33 ± 8.24*	0.0422
Neutral detergent fiber	456.26 ± 14.45	454.9 ± 3.98	0.883
Acid detergent fiber	295.58 ± 9.89	316.64 ± 0.84	0.0959
Crude protein	40.63 ± 2.71	41.09 ± 0.7	0.7899
Crude fat	14.4 ± 0.42***	21.27 ± 0.64***	0.0001
Ammonium nitrogen	0.31 ± 0.03	0.36 ± 0.02	0.0572
pH	4.58 ± 0.04**	4.46 ± 0.01**	0.0086

*p < 0.05; **p < 0.01, ***p < 0.001.

### 3.2 Mycotoxin content test results

The ELISA standard curves for AFT and T2 demonstrated excellent linearity, with R^2^ values of 0.987 and 1.000, respectively. Quantitative analysis revealed significant reductions in mycotoxin levels in the LT group: AFT content decreased (*p* < 0.01), while T2 content showed a more pronounced reduction (*p* < 0.001) (Table 2).

**Table 2:** Mycotoxin content.

Items	LC (ng/mL)	LT (ng/mL)	*p*-value
AFT	1.06 ± 0.07	0.75 ± 0.04	0.0029
T2	17.46 ± 2.01	3.73 ± 1.01	0.0004

### 3.3 Short-chain fatty acid content

As presented in Table 3, lactic acid content demonstrated a substantial increase (*p* < 0.001). Analysis of short-chain fatty acid profiles (Table 4) revealed significant changes: butyric acid content decreased markedly (*p* < 0.01), while isocaproic acid showed a pronounced increase (*p* < 0.001). Other short-chain fatty acids remained statistically unchanged.

**Table 3:** Lactic acid content.

Items	LC (g/kg DM)	LT (g/kg DM)	*p*-value
Lactic acid	20.79 ± 0.26	22.75 ± 0.53	0.0046

*p < 0.05; **p < 0.01, ***p < 0.001.

**Table 4:** Short chain fatty acid content.

Items	LC (μg/mg)	LT (μg/mg)	*p*-value
Acetic acid	11.16 ± 4.37	7.96 ± 1.39	0.1211
Propanoic acid	0.19 ± 0.02	0.17 ± 0.02	0.3274
Iso butyric acid	0.02 ± 0.00	0.02 ± 0.00	0.6671
Butanoic acid	0.24 ± 0.46^**^	0.02 ± 0.00^**^	0.0023
Isovaleric acid	0.012 ± 0.01	0.01 ± 0.00	0.2068
Valeric acid	0.01 ± 0.00	0.01 ± 0.00	0.412
Iso hexanoic acid	0.14 ± 0.03***	0.31 ± 0.03***	<0.0001
Hexanoic acid	0.02 ± 0.01	0.01 ± 0.00	0.3279

*p < 0.05; **p < 0.01, ***p < 0.001.

### 3.4 Microbial diversity analysis of *Pennisetum giganteum* silage

High-throughput sequencing of silage samples on the Illumina platform yielded 2,108,371 quality-filtered bacterial sequences and 2,603,646 fungal sequences after removing low-quality reads. Sequence clustering and taxonomic annotation identified 1,042 operational taxonomic units (OTUs), comprising 340 bacterial and 702 fungal OTUs. Bacterial community analysis revealed several key patterns: rank-abundance curves indicated superior community uniformity in LT samples, while rarefaction curve stabilization confirmed adequate sampling depth and data reliability (Supplementary Figure S1A). Alpha diversity metrics (Chao1, Shannon, and Simpson indices) showed no significant differences between groups (*p* > 0.05), however, it was clearly observed that the differences in the *α*-diversity indices within the LT group were minimal (Supplementary Figures S1B–D). Venn analysis identified 340 shared bacterial OTUs, with 96 and 67 OTUs unique to LC and LT groups, respectively (Supplementary Figure S1E). PCoA revealed distinct clustering patterns, with LT samples showing greater homogeneity and two LT samples significantly diverging from LC samples (Supplementary Figure S1F). Fungal community diversity analysis revealed improved homogeneity in LT samples, as demonstrated by rank-abundance curves, while rarefaction curve stabilization confirmed adequate sampling depth and data reliability (Figure S1A). Alpha diversity metrics, including Chao1, Shannon, and Simpson indices, showed no significant differences between groups (*p* > 0.05) (Supplementary Figures S2B–D). Venn analysis identified 702 shared fungal OTUs, with 419 and 418 OTUs unique to LC and LT groups, respectively (Supplementary Figure S2E). Principal coordinate analysis (PCoA) revealed limited overall differentiation between groups, though LC samples exhibited greater dispersion in the multivariate space (Supplementary Figure S2F).

### 3.5 Microbial composition of *Pennisetum giganteum* silage

**Figure 1:**
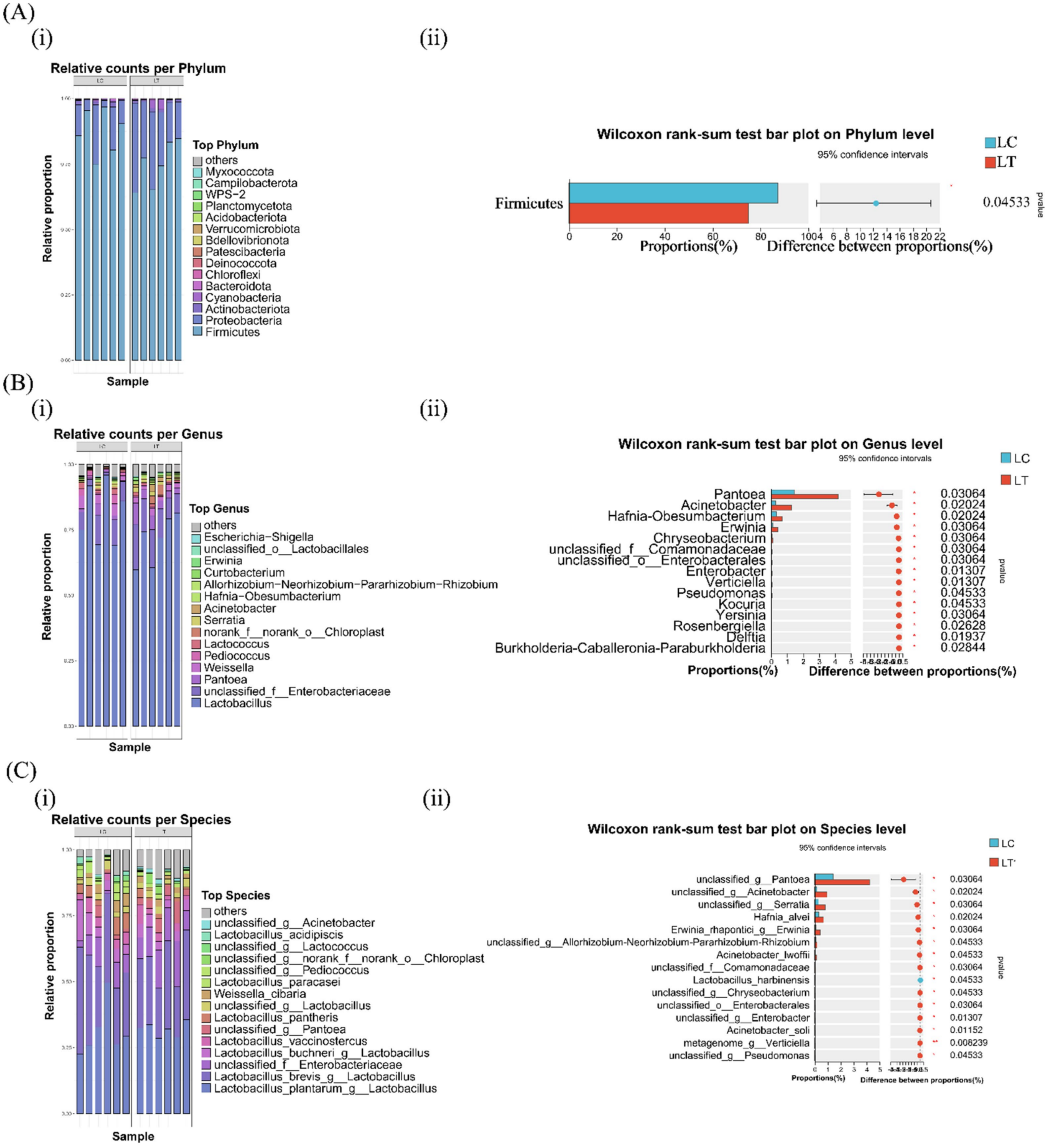
Effects of *Lactobacillus brevis* R-09 additive on the relative abundance of bacterial community in *Pennisetum giganteum* silage at Phylum and Species levels. **(A)** The abundance accumulation map **(i)** of the first 15 gates and the difference histogram at the gate level **(ii)**; **(B)** the abundance accumulation diagram of the first 15 genera and **(i)** the difference histogram of genus level **(ii)**; **(C)** Abundance plot **(i)** and species level difference histogram **(ii)** of the first 15 species.

Bacterial community composition across taxonomic levels is presented in Figure 1. At the phylum level, both LC and LT groups were dominated by *Firmicutes* and *Proteobacteria*, with *Actinobacteriota*, *Cyanobacteria*, and *Bacteroidota* as secondary phyla (Figure 1A i). Notably, *L.brevis* R-09 supplementation significantly reduced *Firmicutes* abundance (*p* < 0.05, Figure 1A ii). At the genus level, *Lactobacillus* dominated both groups, followed by unclassified *Enterobacteriaceae*, *Weissella*, *Pediococcus* and *Pantoea* in LC, and unclassified *Enterobacteriaceae*, *Pantoea* and *Weissella* in LT (Figure 1B i). *L.brevis* R-09 supplementation significantly altered the relative abundance of 15 genera, including *Pantoea*, *Acinetobacter*, and *Pseudomonas* (*p* < 0.05, Figure 1B ii). Species level analysis revealed *Lactobacillus plantarum* and *L.brevis* as dominant species in both groups, with *L.buchneri*, unclassified *Enterobacteriaceae*, and *L.pantheris* as secondary species in LC (Figure 1C i). The additive induced significant changes in 13 species’ abundance (*p* < 0.05), including unclassified *Pantoea* and *Acinetobacter*, while *metagenome_g_Verticiella* showed a marked increase (*p* < 0.01, Figure 1C ii).

**Figure 2:**
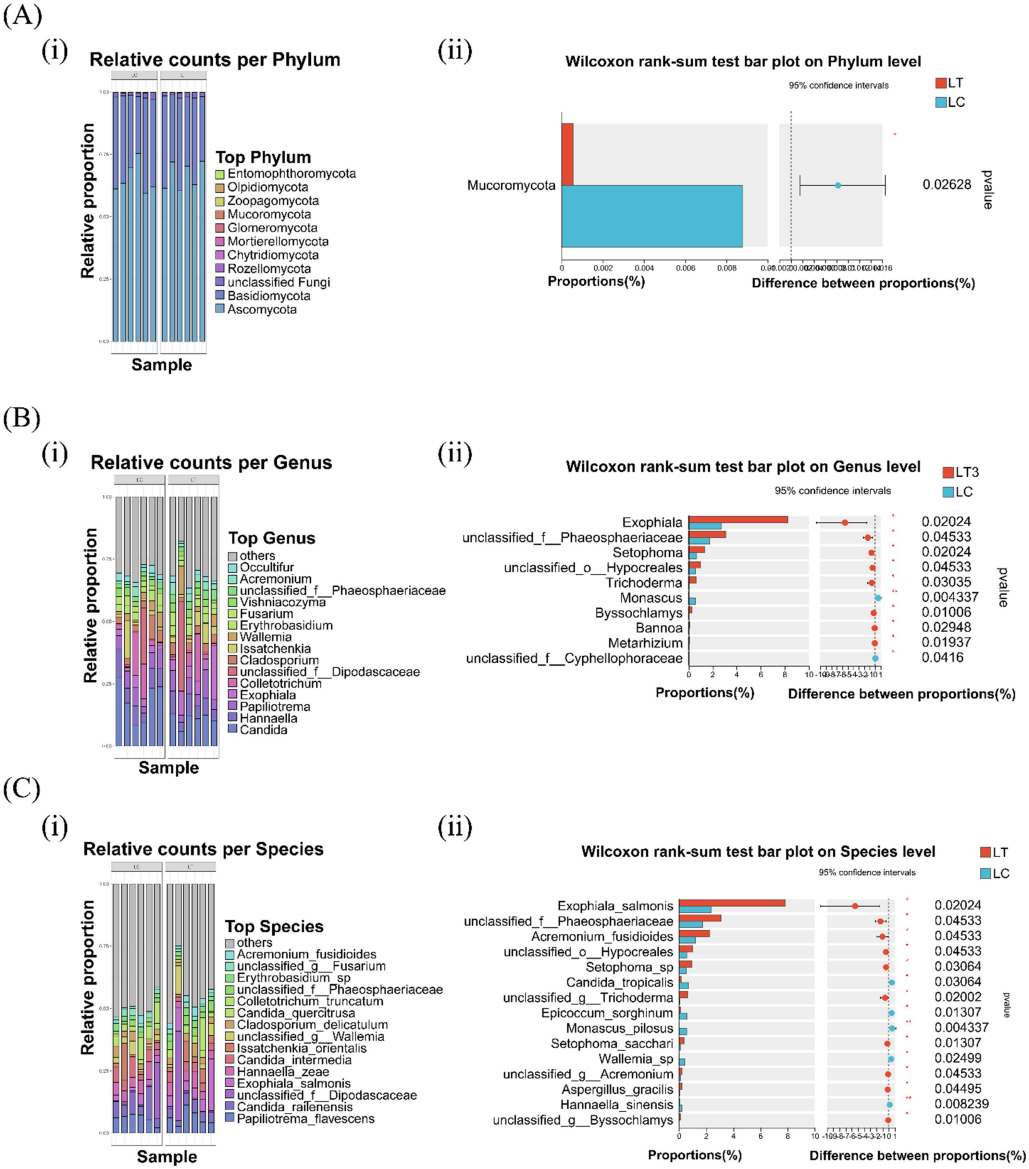
Effects of *Lactobacillus brevis* R-09 additive on the relative abundance of fungal community in *Pennisetum giganteum* silage at phylum, genus and species levels. **(A)** Abundance stacking maps **(i)** and difference histograms **(ii)** of 11 gates; **(B)** the abundance accumulation diagram of the first 15 genera and **(i)** the difference histogram of genus level **(ii)**; **(C)** Abundance plot **(i)** and species-level difference histogram **(ii)** of the first 15 species.

Fungal community composition across taxonomic levels is presented in Figure 2. At the phylum level, *Ascomycota* and *Basidiomycota* dominated both LC and LT, with *L. brevis* R-09 supplementation significantly reducing *Mucoromycota* abundance (*p* < 0.05, Figure 2A i). Genus level analysis revealed *Candida*, *Hannaella*, and *Papiliotrema* as dominant taxa in both groups, though their relative abundances differed between treatments (Figure 2B i). The additive significantly increased the abundance of eight genera, including *Exophiala* and *Trichoderma*, while decreasing unclassified *Cyphellophoraceae* (*p* < 0.05) and *Monascus* (*p* < 0.01, Figure 2B ii). Species level profiling identified *Candida railenensis* and *Papiliotrema flavescens* as dominant species in both groups (Figure 2C i). *L.brevis* R-09 supplementation significantly increased the abundance of ten species, including *Exophiala salmonis* and *Setophoma sacchari*, while decreasing five species, with *Hannaella sinensis* and *Monascus pilosus* showing marked reductions (*p* < 0.01, Figure 2C ii).

### 3.6 Results of LEfSe analysis

**Figure 3:**
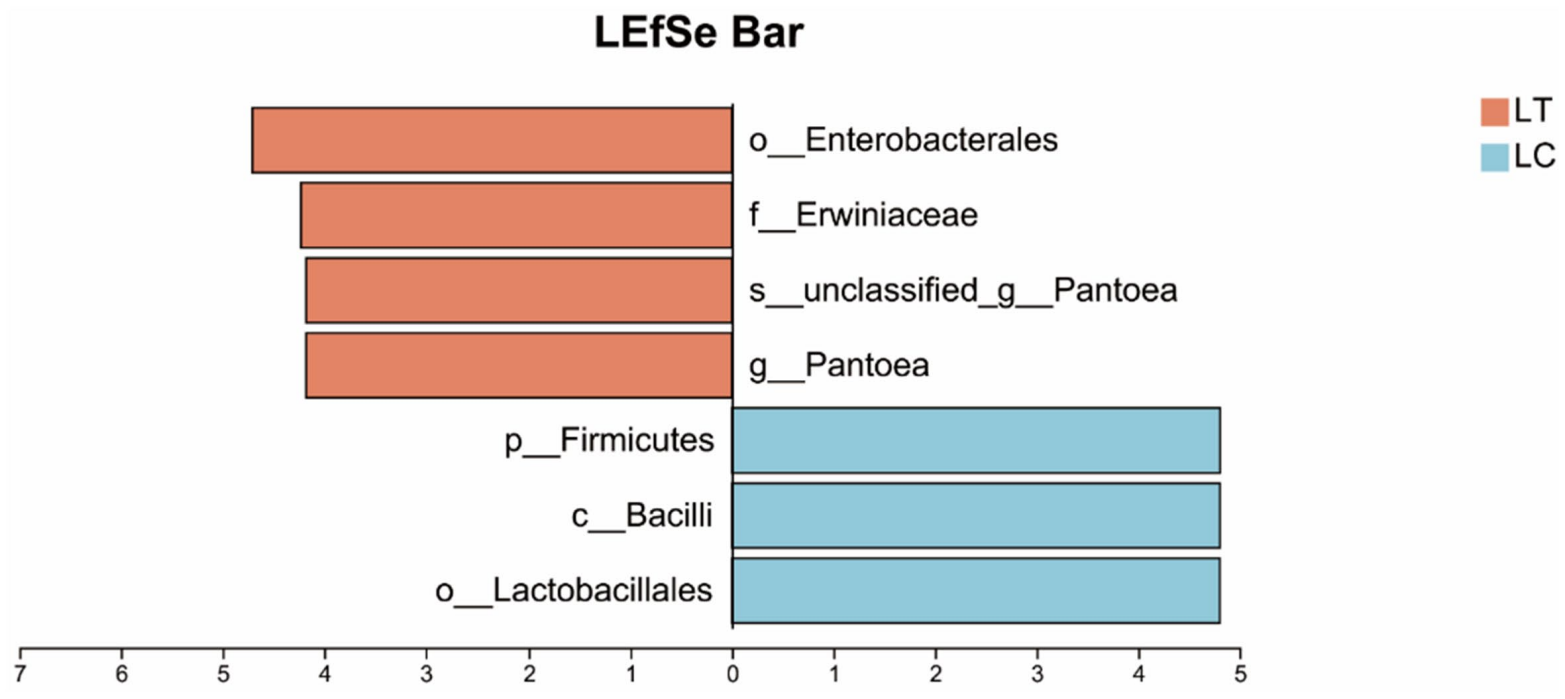
Linear discriminant analysis effect size (LEfSe) of the microbial community of *Pennisetum giganteum* silage based on LDA score threshold > 4.

Using a Linear discriminant analysis (LDA) score threshold > 4 as the criterion for identifying bacterial biomarkers, differential abundance analysis revealed distinct taxonomic patterns between groups: *Pantoea*, unclassified *Pantoea*, *Erwiniaceae* and *Enterobacterales* were significantly enriched in the LT, while *Lactobacillales*, *Bacilli* and *Firmicutes* showed higher relative abundance in the LC (Figure 3).

**Figure 4:**
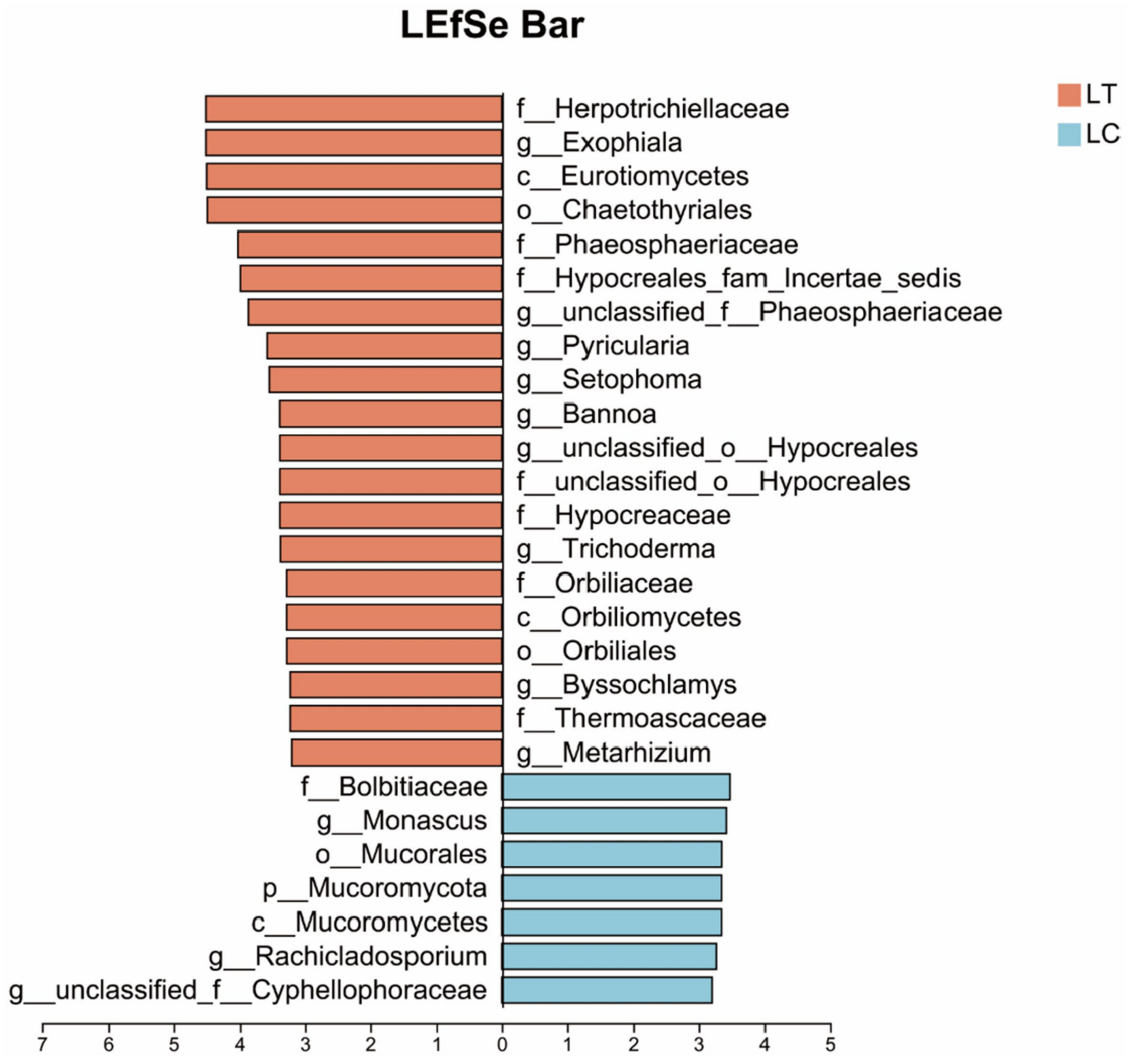
LEfSe of the bacterial community of *Pennisetum giganteum* silage based on the LDA score threshold > 3.

Employing an LDA score threshold > 3 for fungal biomarker identification, significant taxonomic differentiation was observed: *Herpotrichiellaceae*, *Exophiala*, *Eurotiomycetes* and 15 additional taxa were markedly enriched in the LT, while *Bolbitiaceae*, *Monascus*, *Mucorales* and four related taxa showed higher relative abundance in the LC (Figure 4).

### 3.7 Symbiotic network diagram of microorganisms of *Pennisetum giganteum* silage

**Figure 5:**
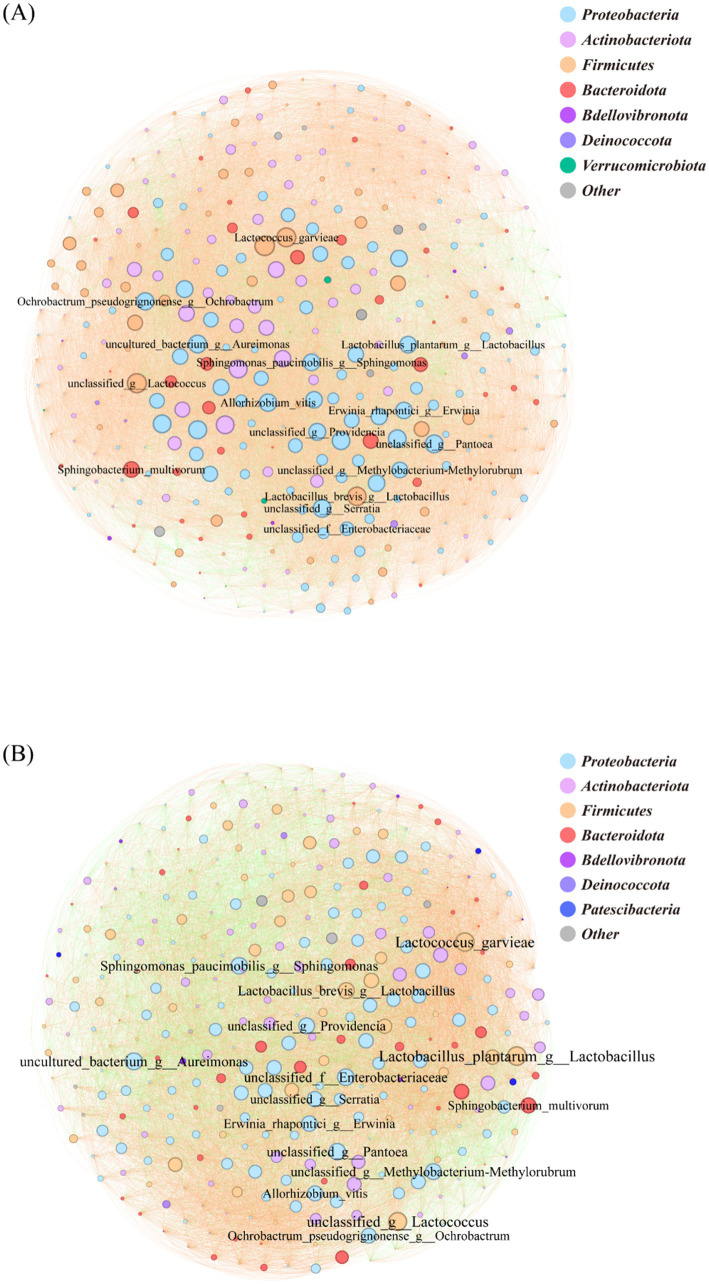
Bacterial correlation network diagram at various levels. The circle represents microbial species-level classification; the color of the circle represents the phylum-level classification; the size of the circle represents the degree of connection at the genus level; the line represents the correlation between two genus-level bacteria; the color of the line represents positive and negative correlations. **(A)**: LT; **(B)**: LC.

The species-level bacterial correlation network in the LC group comprised 356 nodes, with *Proteobacteria* representing the most abundant phylum (38.20%), followed by *Actinobacteriota* (22.75%), *Firmicutes* (19.10%) and *Bacteroidota* (12.64%). Network analysis identified *Weissella cibaria*, *L. brevis* and *Serratia marcescens* among the top 15 most connected species. The network was predominantly characterized by positive correlations (90.94%), with negative interactions representing only 9.06% of total connections (Figure 5B). The LT group’s bacterial correlation network contained 333 nodes, with *Proteobacteria* (41.44%) *Actinobacteriota* (20.12%) and *Firmicutes* (17.42%) as the dominant phyla (Figure 5A). Network topology analysis revealed *L. plantarum* and *L. garvieae* as the most connected species among the top 15 nodes. Compared to LC, the LT network showed a notable shift in interaction patterns, with positive correlations decreasing to 71.3% and negative interactions increasing to 28.7% (Figure 5A).

**Figure 6:**
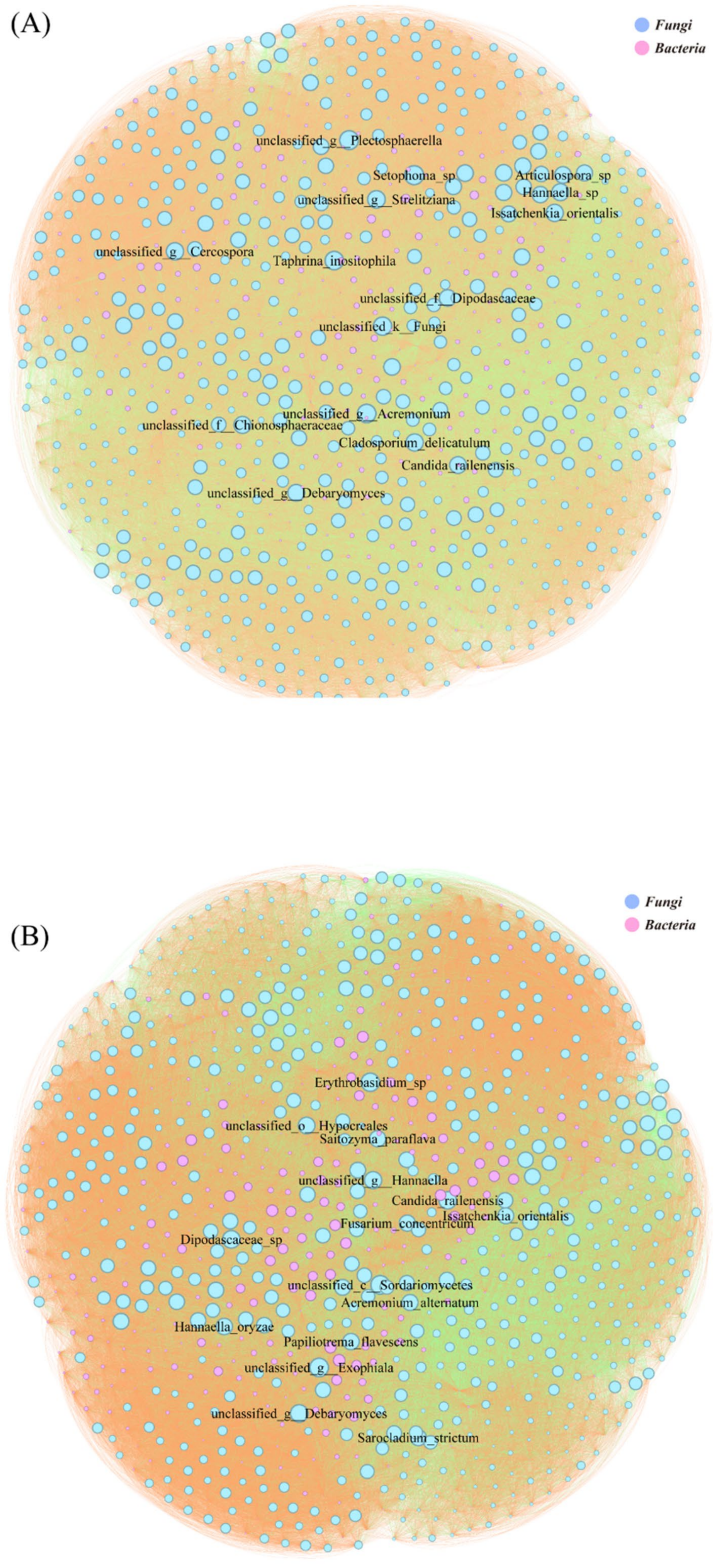
Microbial correlation network diagram of various levels. The circle represents microbial species-level classification; the color of the circle represents the phylum-level classification; the size of the circle represents the degree of connection at the genus level; the line represents the correlation between two genus-level bacteria; the color of the line represents positive and negative correlations. **(A)**: LT; **(B)**: LC.

The species-level microbial correlation network in the LC group comprised 933 nodes, with Fungi (61.84%) and Bacteria (38.16%) as the dominant kingdoms (Figure 6B). Network analysis identified unclassified *Sordariomycetes* and *Exophiala* among the top 15 most connected species. The network exhibited a predominance of positive interactions (69.91%), with negative correlations representing 30.09% of total connections (Figure 6B). The LT group’s microbial correlation network contained 894 nodes, maintaining a similar fungal-bacterial ratio (62.75% Fungi, 37.25% Bacteria) to LC (Figure 6A). Network topology analysis revealed reduced mycobacterial connectivity and identified *Setophoma* sp. and unclassified *Plectosphaerella* among the top 15 most connected species. Compared to LC, the LT network showed a slight increase in negative interactions (33.41%) with corresponding decrease in positive correlations (66.59%) (Figure 6A).

### 3.8 Effects of *Lactobacillus brevis* R-09 on metabolites of *Pennisetum giganteum* silage

Metabolomic analysis of *P. giganteum* silage identified 991 metabolites, categorized into 11 chemical classes, including 134 lipids and lipid-like molecules (HMDB) and 43 phenylpropanoids (Supplementary Figure S3A). KEGG classification revealed 12 compounds across seven categories, predominantly lipids and carbohydrates (Supplementary Figure S3B). Multivariate analysis demonstrated distinct metabolic profiles: principal component analysis showed subtle intergroup differences (Supplementary Figure S4A), while orthogonal partial least squares discriminant analysis revealed more pronounced separation, with LT samples exhibiting greater clustering (Supplementary Figure S4B). Venn analysis identified 1,268 shared metabolites, with 8 LT-specific and 17 LC-specific compounds (Supplementary Figure S4C). Differential metabolite analysis (*p* < 0.05, Vip > 1) identified 464 compounds, including 45 up-regulated and 76 down-regulated metabolites (|log2FC| > 0.05) in LT (Supplementary Figure S4D).

**Figure 7:**
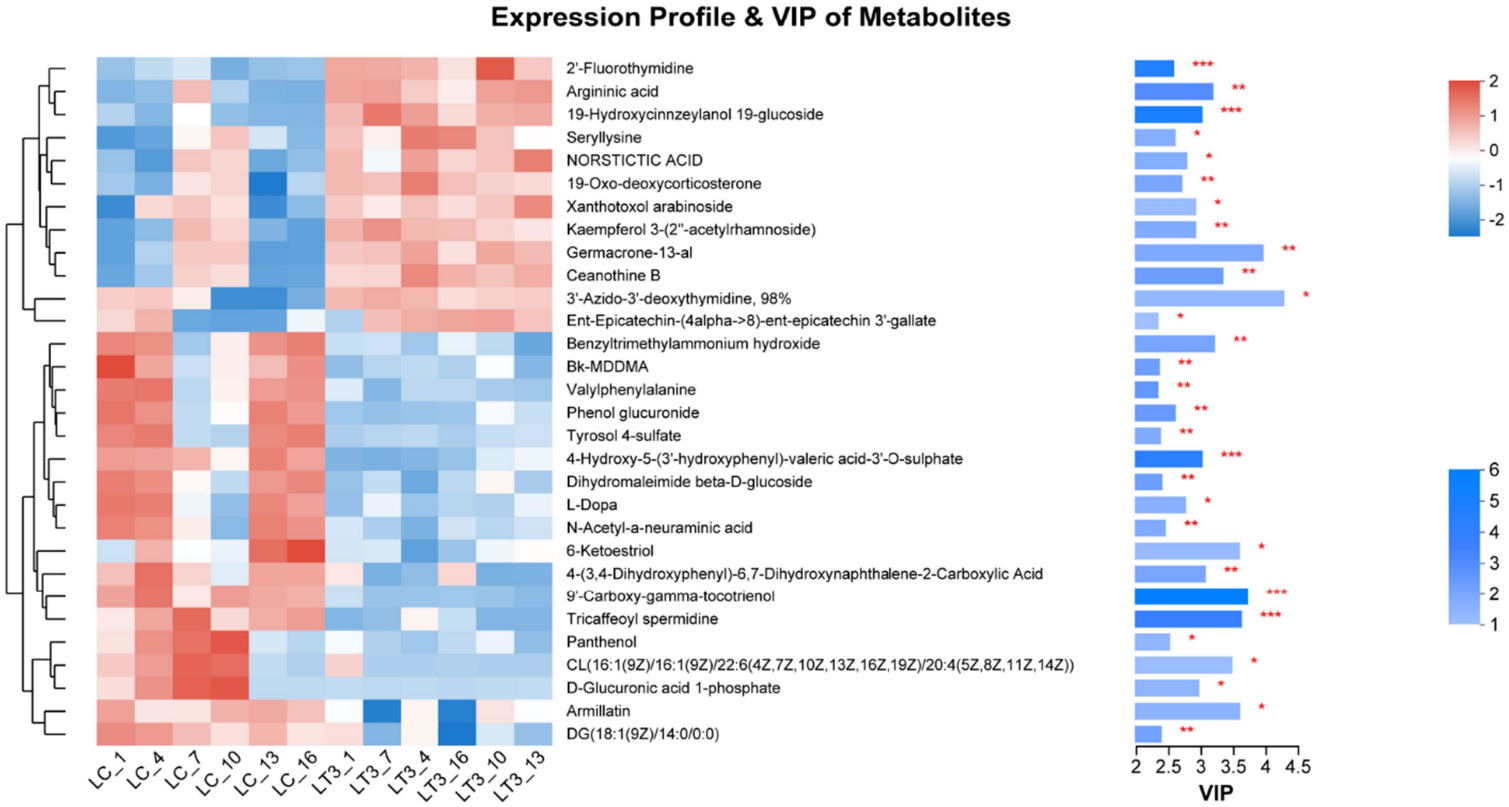
VIP heat map.

Analysis of variable importance in projection (VIP) scores identified the top 30 differentially regulated metabolites following *L. brevis* R-09 supplementation. Notably, 9’-Carboxy-gamma-tocotrienol, 19-hydroxycinnzeylanol-19-glucoside, and three additional metabolites exhibited the most pronounced intergroup differences (*p* < 0.01), highlighting their potential role in mediating the additive’s effects (Figure 7).

### 3.9 KEGG metabolic pathway

**Figure 8:**
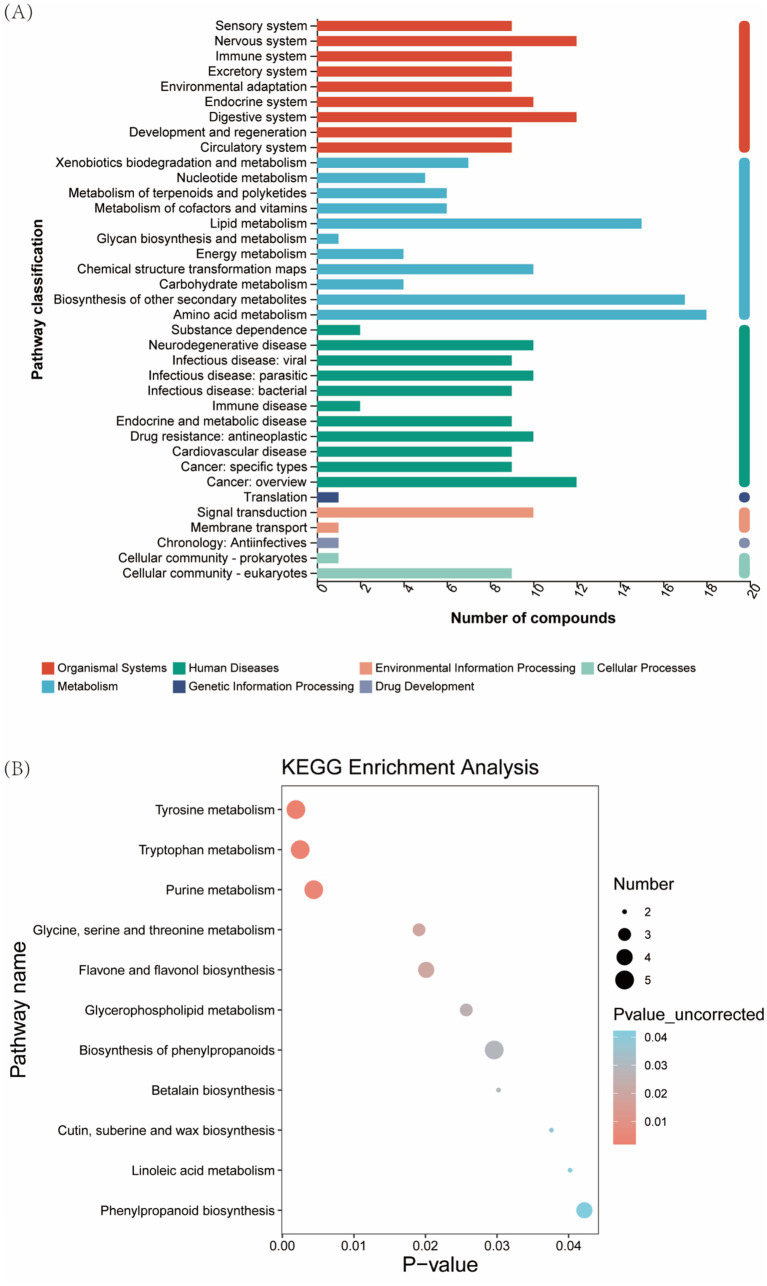
Enrichment analysis of functional pathways in *Pennisetum giganteum* silage. **(A)** KEGG functional pathway, **(B)** KEGG metabolic pathway enrichment analysis.

Metabolites were systematically classified into 7 primary and 37 secondary functional pathways. Secondary pathways, particularly amino acid metabolism, secondary metabolite biosynthesis, and lipid metabolism, demonstrated significant metabolite enrichment. Primary pathway analysis revealed 93 metabolites involved in metabolism, 88 in organismal systems, and 91 in human diseases (Figure 8A). Pathway enrichment analysis identified 11 significantly altered metabolic pathways, including tyrosine metabolism, flavonoid biosynthesis, and glycerophospholipid metabolism, highlighting key biochemical processes influenced by silage fermentation (Figure 8B).

### 3.10 Results of correlation analysis of differential metabolites with dominant microorganisms

**Figure 9:**
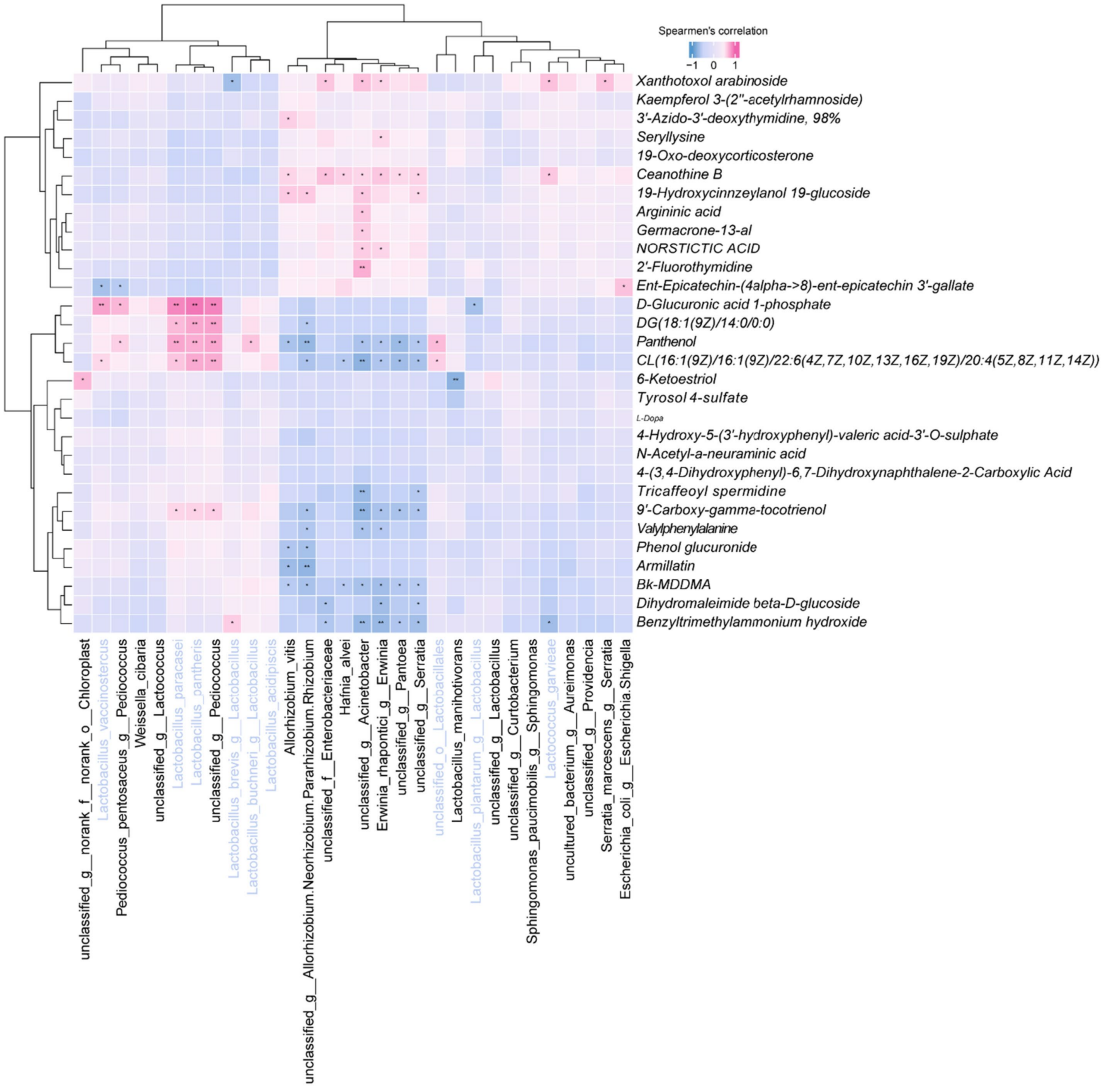
Heatmap of the correlation between the top 30 bacteria at the genus level and the top 30 metabolites at the VIP value.

Correlation analysis revealed significant associations between top 30 Bacteria and specific metabolites (Figure 9). Notably, *L. vaccinostercus* exhibited negative correlations with CL (16:1/16:1/22:6/20:4) and positive correlations with D-glucuronic acid 1-phosphate. *L. paracasei* and *L. pantheris* showed consistent negative correlations with 9′-carboxy-gamma-tocotrienol and specific lipids, while positively correlating with D-glucuronic acid derivatives. *L. brevis* demonstrated unique associations with benzyltrimethylammonium hydroxide and xanthotoxol arabinoside. Cluster analysis indicated similar metabolic modulation patterns among *L. vaccinostercus*, *P. pentosaceus*, *W. sibaria* and related species, suggesting conserved functional roles in shaping the silage metabolome.

## 4 Discussion

Dry matter content, a key indicator of silage nutritional quality (14), showed no significant intergroup differences in this study. This observation may reflect the complex microbial ecology of *P. giganteum* silage and the initial lag phase of *L. brevis* R-09 growth during early fermentation, failure to promptly inhibit dry matter degradation by aerobic bacteria. Notably, both groups exhibited higher dry matter content than previous reports (15), potentially attributable to variations in agronomic factors including climate, soil conditions, harvest timing and nutrient structure of *P. giganteum*. Crude fiber content, inversely related to palatability (16), and crude fat content, a nutritional quality marker (17). In this study, the inclusion of *L. brevis* R-09 in *P. giganteum* silage resulted in a significant reduction in crude fiber content and an increase in crude fat content, thus improving the nutritional quality of the silage. This effect could be attributed to the production of cellulase by *L. brevis* R-09, which facilitates the breakdown of cellulose and hemicellulose. In addition, the acidification of the silage caused by *L. brevis* R-09 may contribute to cell wall acidification, resulting in decreased crude fiber content (18). The increase in crude fat content may be due to the acidified silage environment or to the production of bacteriostatic compounds by *L. brevis* R-09 that suppress the growth of microorganisms that consume crude fat, thereby preserving its content.

pH serves as a critical parameter for evaluating the fermentation quality of silage, and it is widely accepted that high quality silage should have a pH of 4.2 or less (19). In this study, the inclusion of *L. brevis* R-09 helped to lower the pH closer to the desired range, but failed to achieve a pH below 4.2 in both groups. Previous studies, such as Guyader (20), have shown that the optimum moisture content for silage is between 65 and 70%, as deviations from this range can inhibit the growth of LAB. Additionally, the inherent leaf nutrient composition and specific water content of *P. giganteum* likely influenced pH stability, potentially explaining the inability to reach the desired pH range (17). During the silage process, short-chain fatty acids play an important role in improving fermentation quality and reducing protein hydrolysis (21). Butyric acid, a type of short-chain fatty acid, is produced by butyric acid-producing bacteria during protein degradation, resulting in odor production and a decrease in feed palatability and nutritional value. Therefore, a higher butyric acid content indicates poorer fermentation quality of silage (22). In this study, the addition of *L. brevis* R-09 significantly reduced the butyric acid content in *P. giganteum* silage, surpassing the results of the aforementioned study. The significant reduction in butyric acid content markedly enhanced the fermentation quality of *P. giganteum* silage, a finding corroborated by sensory evaluation results demonstrating improved organoleptic properties in the treated silage. Furthermore, the observed increase in iso-hexanoic acid content, a marker of improved carbohydrate retention (23), suggests enhanced sugar preservation in the treated silage. These modifications in organic acid profiles demonstrate the potential of *L. brevis* R-09 for optimizing the fermentation characteristics of *P. giganteum* silage.

Wayne (24) found that the predominance of certain microbial species tends to determine the stability of phytoplasmas, and a higher proportion of dominant species leads to a more homogeneous overall microbial community. Compared to previous studies (15), although there was no significant difference in alpha diversity between the two groups in our study, both groups had similar alpha diversity values as previously reported. This similarity confirms the presence of dominant microbial species in both groups, which contributes to the homogenization of the microbial community. PCoA revealed minimal differentiation between LT and LC groups, though LT samples treated with *L. brevis* R-09 exhibited greater clustering and uniformity. This pattern of enhanced microbial community homogeneity in LAB-supplemented silage is consistent with previous observations by Liu (25) and Hao (26), suggesting a characteristic response to lactic acid bacteria inoculation. The observed diversity patterns likely result from rapid LAB proliferation during the aerobic fermentation phase, establishing microbial dominance in both treatments. However, variability in epiphytic LAB concentrations within the LC group may have led to differential competitive interactions between LAB and undesirable microorganisms, contributing to inter-sample variation. This phenomenon underscores the importance of initial epiphytic LAB populations in shaping microbial community dynamics during silage fermentation.

High-throughput sequencing analysis identified Firmicutes and Proteobacteria as the dominant phyla in *P. giganteum* silage, consistent with Wang’s findings (27). Notably, the significant reduction in Firmicutes abundance in the LT group, without corresponding changes in *Proteobacteria* (which includes pathogenic gram-negative species (28)), suggests potential antagonistic interactions between *L. brevis* R-09 and other Firmicutes members, aligning with Keshri’s observations (29). At the genus level, Lactobacillus dominated both groups, playing a pivotal role in silage fermentation quality (30). This dominance may reflect favorable environmental conditions in Yunnan that promote LAB proliferation, resulting in substantial epiphytic LAB populations on *P. giganteum*. The presence of *Pantoea*, previously observed in silage systems (31), warrants further investigation regarding its functional role. Other notable genera included *Pediococcus* an early-stage lactic acid producer and *Weissella* a heterofermentative bacterium associated with acetic acid production (32). While *Enterobacter* and *Yersinia* showed increased relative abundance in the LT group, their overall levels remained low. This pattern may reflect incomplete acidification due to *L. brevis* R-09’s antagonistic effects on *Firmicutes*, potentially limiting the establishment of optimal inhibitory conditions. Species-level analysis revealed *L. plantarum* and *L. brevis* as dominant species, consistent with Yan’s findings (33) and aligned with the widespread use of these species as silage inoculants, Such as *L.plantarum* (34), *L.buchneri* (35) and *L.brevis* (36).

Fungal community analysis revealed *Ascomycota* and *Basidiomycota* as the dominant phyla in *P. giganteum* silage, consistent with Peng’s findings (37). These phyla encompass molds and yeasts that contribute to aerobic spoilage and nutritional degradation during fermentation (38). Notably, *L. brevis* R-09 supplementation significantly reduced *Mucoromycota* abundance, a known silage spoilage pathogen (39). Genus-level profiling identified *Candida* and *Hannaella* as dominant taxa, with Saccharomycetales members (*Candida*, unclassified *Dipodascaceae*, *Hannaella*) representing key silage-associated fungi (40). While most epiphytic fungi decline during ensiling, *Candida* facultative anaerobic nature explains its persistence (39). The detection of *Papiliotrema*, potentially involved in acetic acid metabolism, aligns with Hou’s observations of its inhibitory effects on acetic acid fermentation (41). *L.brevis* R-09 supplementation significantly decreased the abundance of potentially pathogenic *Cyphellophoraceae* (42) and *Monascus* a toxin-producing mold associated with aerobic spoilage (43). However, increased abundance of other potentially detrimental genera (*Exophiala*, *Byssochlamys*, *Trichoderma*) may reflect selective inhibition by *L. brevis* R-09 metabolites. Species-level analysis identified *Papiliotrema flavescens* and *Candida railenensis* as highly abundant, with significant reductions in *Wallemia* sp., *Epicoccum sorghinum* (a plant pathogen (44)), and *Candida tropicalis* in the LT group. The observed inter-sample variability in fungal populations likely reflects inherent differences in epiphytic communities (45), highlighting the complex microbial dynamics in silage systems.

Correlation network analysis revealed distinct microbial interaction patterns between treatment groups, with network topology reflecting competitive and cooperative dynamics (46). The LT exhibited reduced positive correlation rates compared to LC, suggesting intense competition between *L. brevis* R-09 and epiphytic microorganisms. This competitive pressure may explain the enhanced sample homogeneity in the LT group, though it potentially limits additive efficacy, which could be mitigated through substrate nutrient optimization or increased inoculum dosage. These findings contrast with Zhao observations of increased cooperation during late fermentation (15), possibly indicating that the LC group had reached a stable fermentation state with limited temporal microbial community changes. Network complexity analysis aligned with Bai findings (47), showing reduced node numbers and simplified topology in the LT group, characteristic of high-quality fermentation. Within the bacterial network, Lactobacillus spp. demonstrated increased connectivity in the LT group, reflecting enhanced competitive interactions both between Lactobacillus and other genera, and within Lactobacillus populations. The fungal-bacterial network analysis revealed a shift from mold-dominated interactions in LC to yeast-dominated dynamics in LT, consistent with observed reductions in mycotoxin content. This pattern suggests effective mold inhibition by *L. brevis* R-09, with yeasts emerging as the primary competitive flora, potentially contributing to improved silage quality through reduced mycotoxin production.

Silage fermentation represents a complex biochemical process involving diverse microbial communities and resulting in extensive metabolic transformations (28). In this investigation, we identified 464 differentially abundant metabolites, surpassing previous reports (48), demonstrating the substantial metabolic impact of *L. brevis* R-09 supplementation. Consistent with Hu findings (48), orthogonal partial least squares discriminant analysis revealed distinct clustering patterns in LT samples, indicating microbial community modulation and metabolic convergence induced by *L.brevis* R-09. The observed intergroup differences in metabolite correlations align with Amaral et al.’s observations (49), highlighting treatment-specific metabolic network restructuring. Among the top 30 VIP-ranked metabolites, several exhibited notable biological activities: 3′-azido-3′-deoxythymidine demonstrated antibacterial properties (50), while norstictic acid showed broad-spectrum antimicrobial activity against various pathogens (51) The negative correlation between L-dopa (a phytotoxic compound (52)) and *L. brevis* R-09 treatment suggests potential detoxification effects. While the functional roles of other high-VIP metabolites remain to be fully elucidated, their differential abundance patterns indicate significant metabolic restructuring in response to *L. brevis* R-09 supplementation, warranting further investigation into their potential roles in silage fermentation dynamics and quality.

Secondary metabolic pathway analysis revealed significant enrichment of amino acid metabolism, lipid metabolism, and secondary metabolite biosynthesis in silage samples. These pathways play crucial roles in supporting microbial growth and reflect active catabolic processes during fermentation (53). Notably, the absence of amino acid metabolism inhibition, typically observed in silage systems (32), represents a distinctive feature of this study. Pathway enrichment analysis identified significant involvement of flavonoid biosynthesis, associated with plant stress resistance (54), and betaine metabolism, potentially offering disease prevention benefits (55). Importantly, metabolic pathways with potential environmental or safety concerns, including alkaloid biosynthesis (56) and methane metabolism (57), showed no significant enrichment. These findings suggest that *L. brevis* R-09 supplementation does not induce potentially hazardous metabolic pathways, supporting its safety profile as a silage additive.

## 5 Conclusion

The supplementation of *Lactobacillus brevis* R-09 as a silage additive in *Pennisetum giganteum* enhanced crude fat content while reducing crude fiber levels and mycotoxin concentrations. It promoted microbial community homogeneity without compromising overall diversity, selectively reducing mold abundance and their ecological influence. Metabolomic analysis revealed treatment-specific metabolic profiles associated with LAB activity, indicating a more controlled and uniform fermentation process. These findings collectively demonstrate the potential of *L. brevis* R-09 as an effective silage additive for optimizing the fermentation quality and nutritional value of *P. giganteum* silage.

## Data Availability

The datasets presented in this study can be found in online repositories. The names of the repository/repositories and accession number(s) can be found below: https://www.ncbi.nlm.nih.gov/, PRJNA1266599.
