# Supplementary material for: Effects of Lactobacillus brevis additives on nutrient composition, fermentation quality, microflora structure and metabolites of Pennisetum giganteum silage
Source: Front Vet Sci. 2025 Jul 23;12:1635386. doi: 10.3389/fvets.2025.1635386 (PMC12325029; doi:10.3389/fvets.2025.1635386)
Supplement: Supplementary file 1 [file Supplementary_file_1.docx]

Supplementary files

**
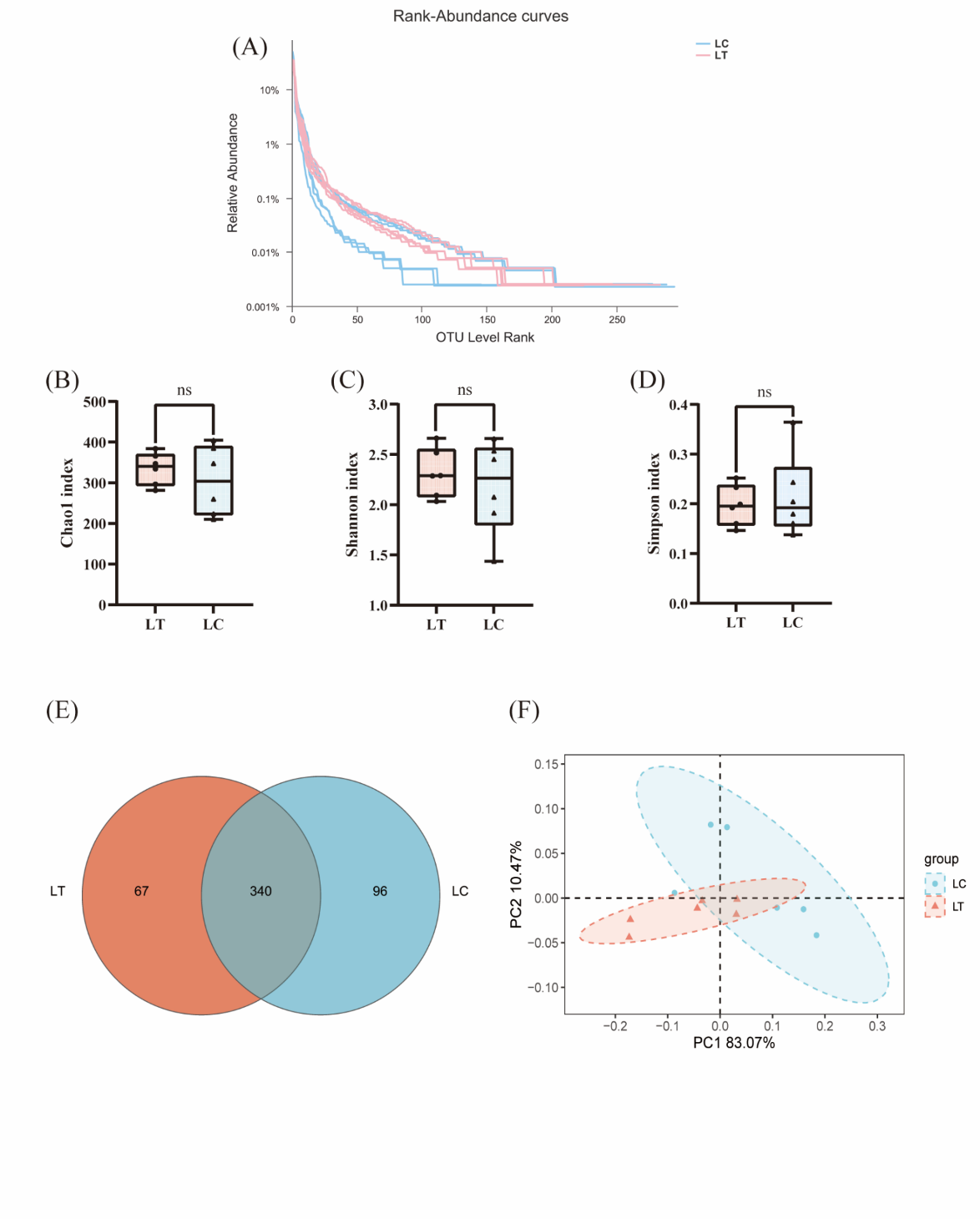
**

Supplementary **Figure** S1. Effects of L. brevis R-09 on bacterial community diversity in P. giganteum silage. (A) Rank-abundance curves; (B) Chao1 index; (C) Shannon index; (D) Simpson index; (E) Venn diagram of OUT; (F) PCoA score plot.

**
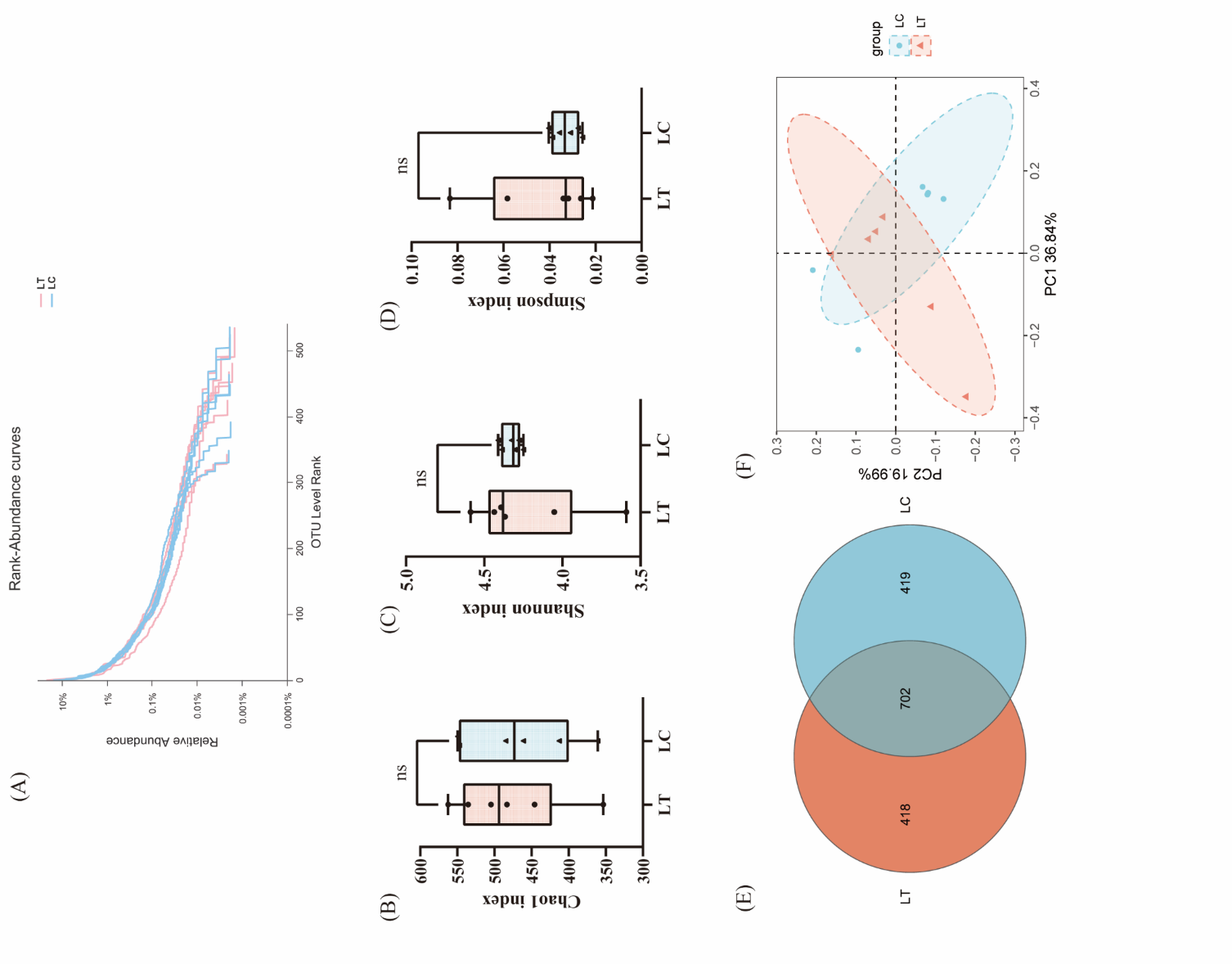
**

Supplementary **Figure** S2. Effects of L. brevis R-09 on fungal community diversity in P. giganteum silage. (A) Rank-abundance curves; (B) Chao1 index; (C) shannon index; (D) Simpson index; (E) Venn diagram of OTU; (F) PCoA score plot.

**
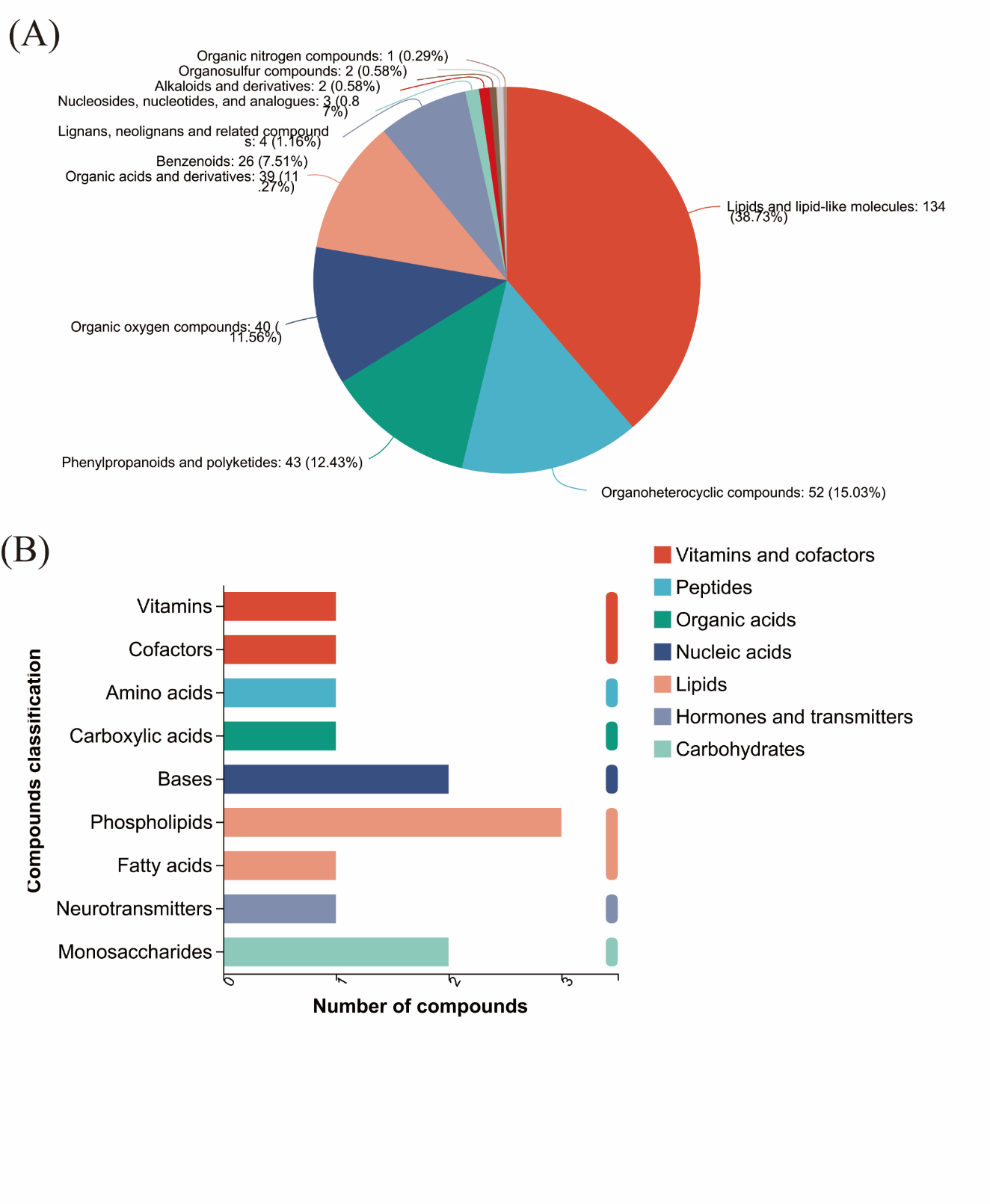
**

Supplementary **Figure** **S3.** Metabolite composition. (A) HMDB compound classification; (B) KEGG compound classification.

**
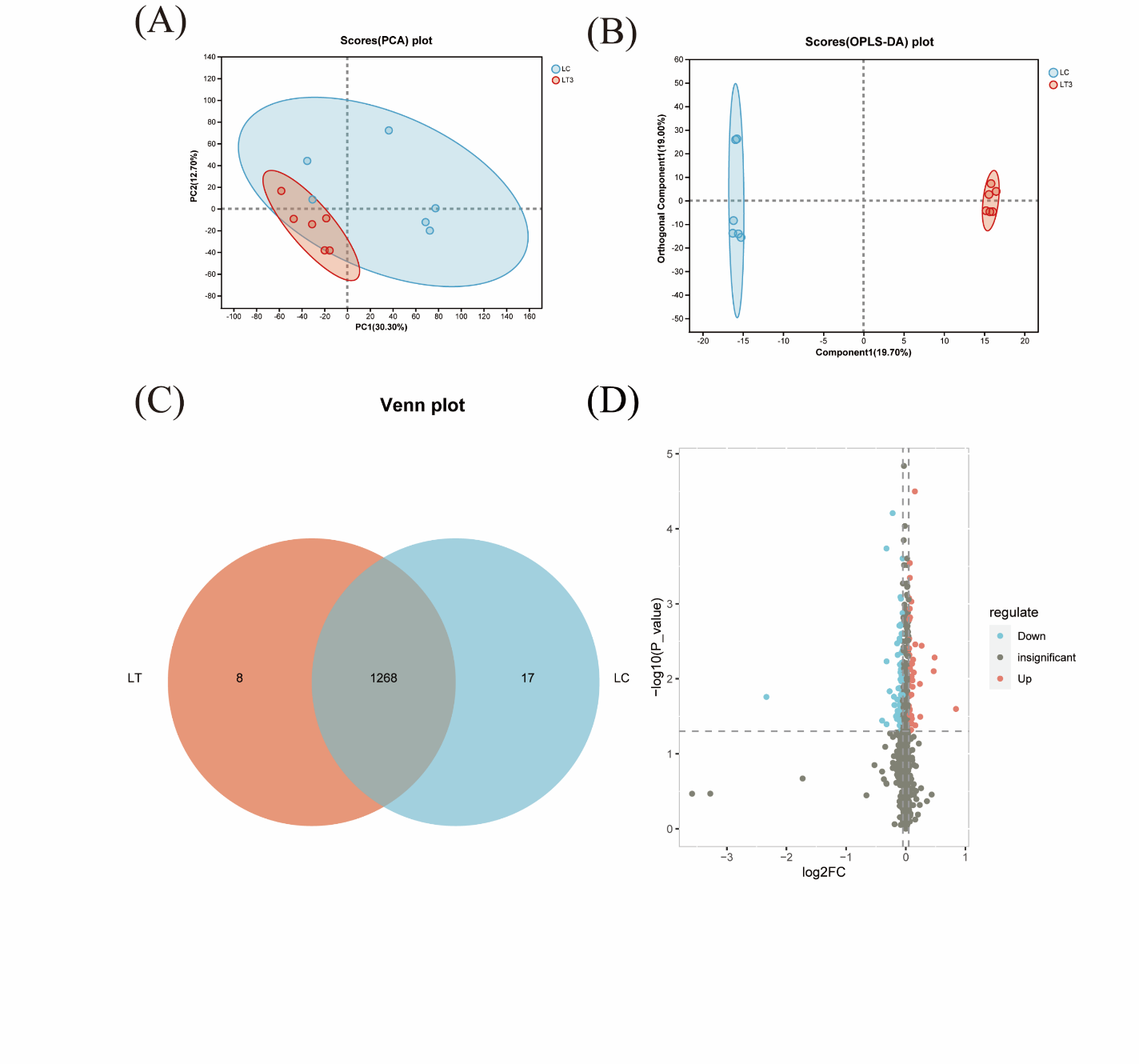
**

Supplementary **Figure** **S4.** Effect of Lactobacillus brevis R-09 additives on metabolites. (A) Principal component analysis; (B) Orthogonal partial least squares discriminant analysis; (C) Wayne diagram; (D) Volcano map.
