# Supplementary material for: Effects of Lactobacillus brevis additives on nutrient composition, fermentation quality, microflora structure and metabolites of Pennisetum giganteum silage
Source: Front Vet Sci. 2025 Jul 23;12:1635386. doi: 10.3389/fvets.2025.1635386 (PMC12325029; doi:10.3389/fvets.2025.1635386)
Supplement: Supplementary file 2 [file Table_1.docx]

| **Item** | **Score** | **High quality** | **Good** | **General** | **Inferior** |
| --- | --- | --- | --- | --- | --- |
| Color | 20 | Turquoise or yellowish green（14~20） | Yellowish brown（8~13） | Brown（1~7） | Dark brown（0） |
| Smell | 25 | Sour aroma（18~25） | Wine aroma（9~17） | Acrid and sour taste（1~8） | Rotten and moldy smell（0） |
| Structure | 10 | Soft but not sticky（8~10） | Loose and soft without stickiness（4~7） | Slightly sticky（1~3） | Sticky agglomeration（0） |
| Moisture content | 20 | To be moist but not to form droplets（14~20） | Tight pressure can form water droplets（8~13） | Tight pressure, there is water outflow（1~7） | Dry or grasp see（0） |
| pH | 25 | 3.4~3.8（18~25） | 3.9~4.1（9~17） | 4.2~4.7（1~8） | Above 4.8（0） |
| Score | | 76~100 | 51~75 | 26~50 | 0~25 |
| Grade | | High quality | Good | General | Inferior |

Supplementary Table S1. Silage sensory evaluation table.
