# Supplementary material for: Effects of Lactobacillus brevis additives on nutrient composition, fermentation quality, microflora structure and metabolites of Pennisetum giganteum silage
Source: Front Vet Sci. 2025 Jul 23;12:1635386. doi: 10.3389/fvets.2025.1635386 (PMC12325029; doi:10.3389/fvets.2025.1635386)
Supplement: Supplementary file 3 [file Table_2.docx]

Supplementary Table S2. *P.giganteum* silage sensory evaluation table.

| **Item** | **LC1** | **LC2** | **LC3** | **LT1** | **LT2** | **LT3** |
| --- | --- | --- | --- | --- | --- | --- |
| Color | 11 | 12 | 8 | 19 | 19 | 20 |
| Smell | 8 | 10 | 5 | 19 | 21 | 20 |
| Structure | 9 | 9 | 9 | 9 | 9 | 9 |
| Moisture content | 8 | 9 | 8 | 9 | 8 | 8 |
| pH | 5 | 5 | 5 | 4 | 4 | 4 |
| Score | 41 | 45 | 35 | 60 | 61 | 61 |
| Grade | General | General | General | Good | Good | Good |
